# Ectopic thyroid tissue accompanied by distinct blood supply and lymphoid tissue within the carotid sheath - a case report

**DOI:** 10.1097/MD.0000000000051110

**Published:** 2026-10-16

**Authors:** Min Chen, Liu Yang, Wen Li, Yan Gao

**Affiliations:** a Department of Otorhinolaryngology Head and Neck Surgery, West China Hospital, Sichuan University, Chengdu, China.

**Keywords:** blood supply, carotid sheath, distinct, ectopic thyroid tissue, lymphoid tissue

## Abstract

**Rationale:**

Ectopic thyroid tissue (ETT) is not extremely rare, but it may be overlooked in clinical practice because of its frequently asymptomatic presentation, lack of urgency for diagnosis, and limitations in diagnostic techniques. We report a case of ETT within the carotid sheath and emphasize its distinct blood supply and accompanying lymphoid tissue, which may improve our understanding of the relationship among the ETT, associated blood supply, and lymphoid tissue from an embryological perspective.

**Patient concerns:**

A 42-year-old male was found to have an asymptomatic mass on the right side of his neck during a routine physical examination.

**Diagnoses:**

Color Doppler ultrasound demonstrated a thyroid gland in the normal location. The mass measured 1.3 cm × 0.9 cm × 0.5 cm in dimensions with well-defined margins, had a similar echotexture to that of the thyroid gland, and a very short venous trunk draining directly into the internal jugular vein.

**Interventions:**

The patient underwent surgical excision of the mass. Histopathological examination after excision confirmed ETT with eminent vasculature and adjacent lymphoid tissue.

**Outcomes:**

The postoperative course was uneventful. The patient has been followed up for 5 years, during which neither recurrence nor thyroid dysfunction was observed.

**Lessons:**

ETT can occur within the carotid sheath even in the presence of a normally located thyroid gland. A distinct vascular supply may represent a useful imaging clue for its diagnosis. Further studies are warranted to investigate the potential embryological association among ETT, its vasculature, and adjacent lymphoid tissue during thyroid descent.

## 1. Introduction

Ectopic thyroid tissue (ETT) is not extremely rare. In addition to its normal anatomical location as the thyroid gland in the pre-tracheal region of the neck, it has also been reported at unusual sites, including the anterior mediastinum, adrenal gland, mandible, and even vertebral bodies.^[1]^ The diagnosis and treatment remain challenging in clinical practice. Thyroid scintigraphy and other imaging modalities may aid in the diagnosis, whereas cytological or histopathological examination is generally required for definitive confirmation. The management strategy depends on the anatomical location, associated symptoms, thyroid function, and the presence or suspicion of malignancy. We report a rare case of ETT within the carotid sheath, with a distinct vascular supply and adjacent lymphoid tissue.

## 2. Case report

A 42-year-old male was admitted to the hospital on March 29, 2021 because a mass was found on the right side of his neck during a routine physical examination. Fiberoptic laryngoscopy showed no abnormalities in the pharynx and larynx. Color Doppler ultrasound revealed that the thyroid gland was normal in size and location, homogeneous in echotexture, and normal in morphology. An isoechoic mass 1.3 cm × 0.9 cm × 0.5 cm in dimensions was found closely related to the internal jugular vein (IJV) with well-defined margins. Punctate and linear Doppler flow signals were visible inside and around the mass (Fig. 1A and B). Contrast-enhanced CT showed a mass with a short-axis diameter of approximately 0.5 cm located anteromedially to the IJV and a prominent short venous trunk, corresponding to the vessel identified on ultrasonography, draining into the IJV. The attenuation and enhancement patterns of the mass was similar to those of the normal thyroid gland (Fig. 1C and D).

**Figure 1.**
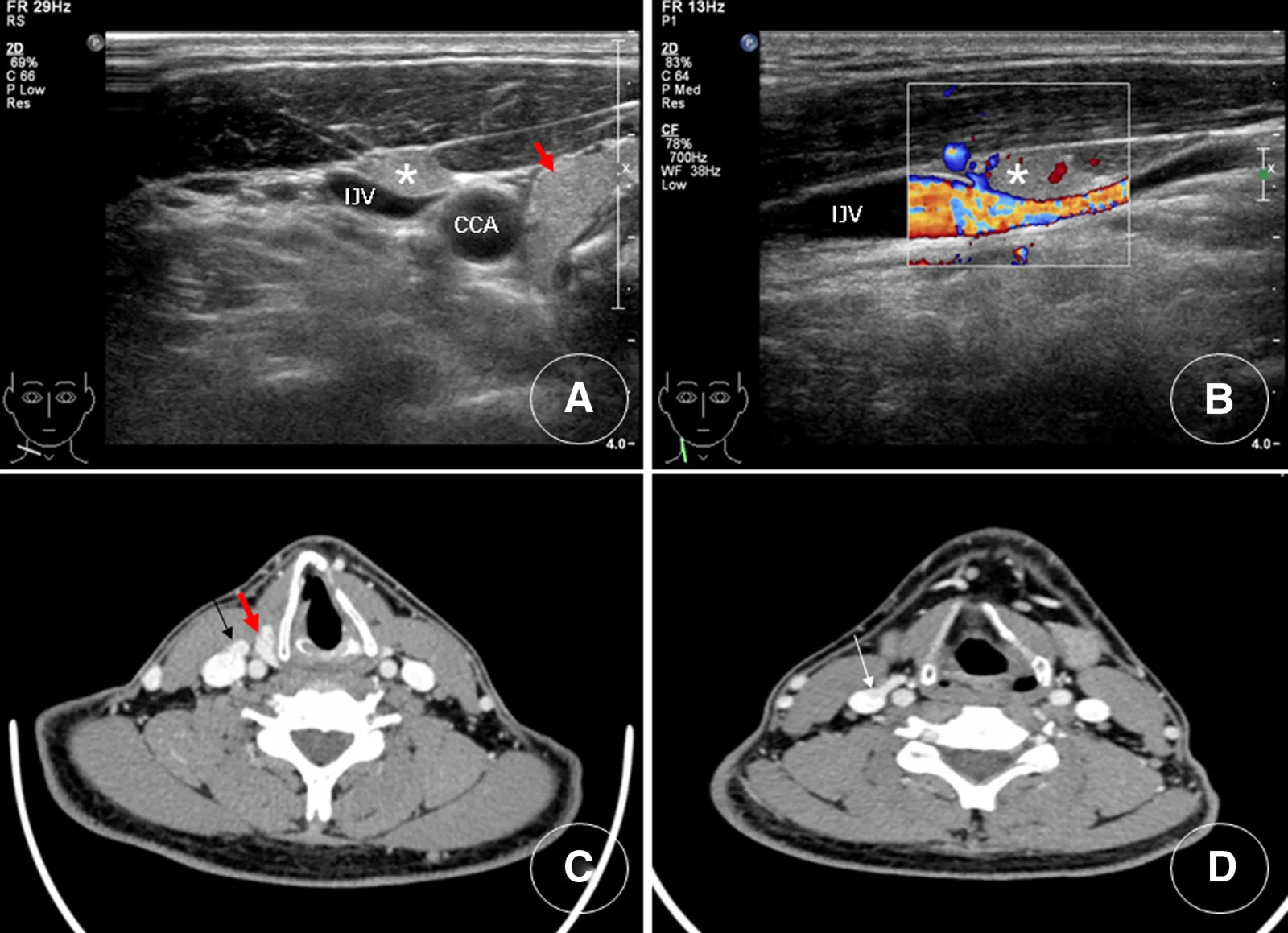
ETT in the carotid sheath. (A) Color Doppler ultrasound showed that the ETT was similar in ultrasound properties to that of a normal thyroid gland; (B) The venous return from the ETT to the internal jugular vein was through a short trunk; (C) Enhanced CT demonstrated the ETT and right upper lobe of the normal thyroid gland; (D) The prominent short vein trunk connected the ETT and IJV. (asterisk* or black arrow: ETT; red arrow: normal thyroid gland; white arrow: short vein trunk). CCA=common carotid artery, ETT = ectopic thyroid tissue, IJV = internal jugular vein.

The mass was surgically excised under general anesthesia. After opening the carotid sheath during the operation, it was found that the mass was spindle-shaped, located between the medial side of the IJV and the right side of the infrahyoid strap muscle (SHM). A distinct small feeding artery, rather than a branch of the superior thyroid artery, originated from the external carotid artery near the carotid bifurcation on the right side. The small artery gave off smaller branches to supply the SHMs along its descending course. The vein of the mass, approximately 1 mm long and 3 mm in diameter, directly drained into the IJV through a short trunk. In addition, a small aggregate of loose connective tissue with clusters of small lymph node-like nodules about 1 to 2 mm in dimension was also found to accompany the mass (Fig. 2A and B). This phenomenon was unusual, because the small amount of lymph nodes and connective tissue was different from the scattered lymph nodes in the lateral neck levels II to IV in gross appearance; instead, it was more like lymph nodes in the central compartment zone.

**Figure 2.**
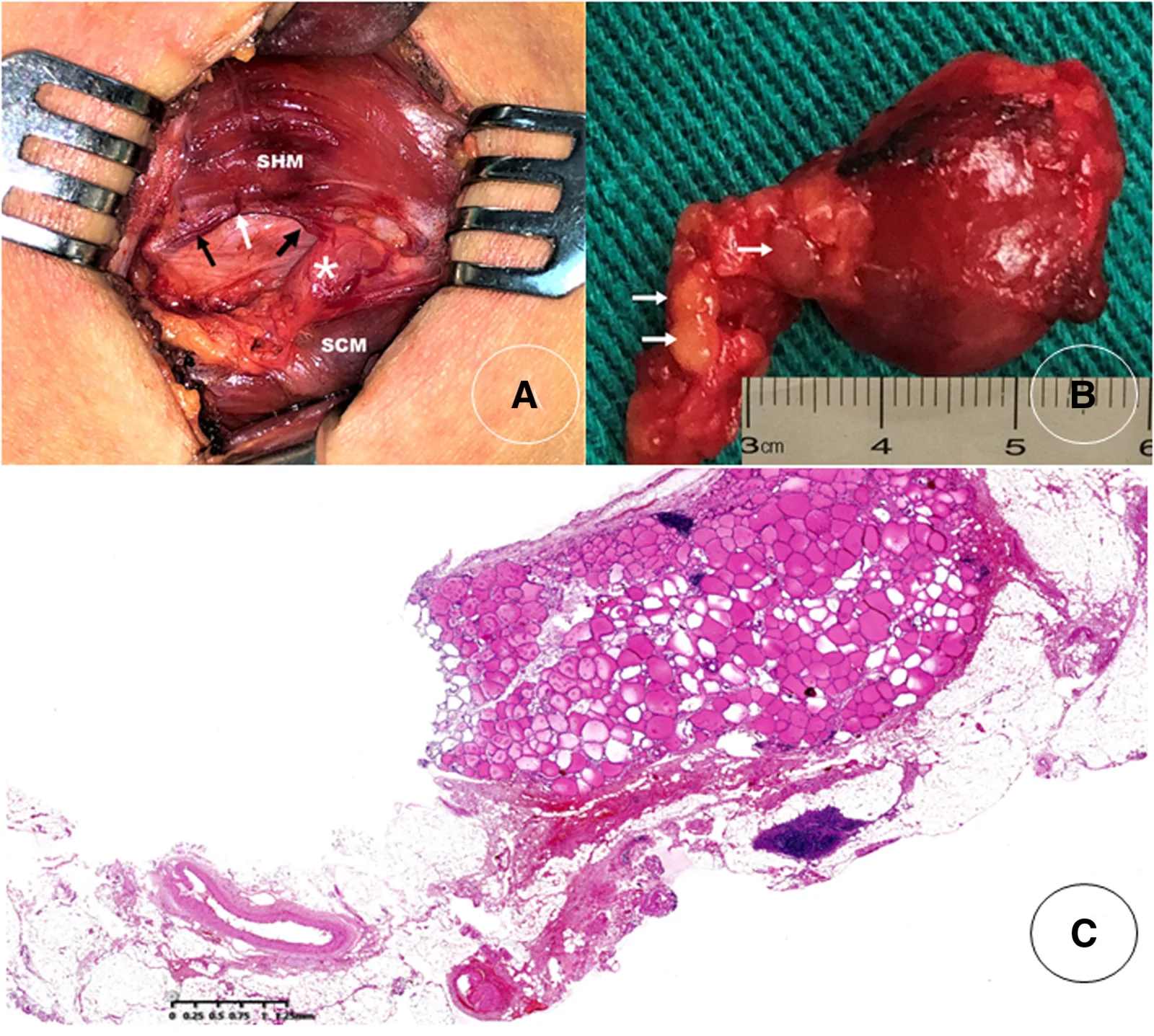
Operation field and histology. (A) The ETT had distinct arterial blood supply and gave off its branches to supply the infrahyoid strap muscle along its way to the ETT; (B) ETT with loose connective tissue and lymph node cluster; (C) The histopathological results revealed ectopic thyroid tissue with surrounding fibrous connective tissue, blood vessels, and non-hyperplastic lymph nodes. (Hematoxylin-eosin staining, HE, original magnification, ◊15; asterisk: ETT; black arrow: artery to the ETT; white arrow: lymph nodes). ETT = ectopic thyroid tissue, SCM = sternocleidomastoid muscle.

Postoperative histopathology confirmed that the mass was histologically thyroid tissue containing prominent blood vessels, with non-hyperplastic lymphoid tissue inside and outside the mass in the same connective tissue capsule (Fig. 2C). The diagnosis of an ectopic thyroid gland was thus established. The cluster of lymph nodes accompanying the ETT showed neither metastasis nor significant reactive hyperplasia or lymphoid follicular formation. The lymph nodes harvested in the lateral neck demonstrated mild to moderate reactive hyperplasia of lymphoid tissue. The color Doppler ultrasound of the orthotopic thyroid gland and thyroid function were within normal limits during the 5-year follow-up time.

## 3. Discussion

ETT is not extremely rare; its diagnosis and management can be challenging in clinical practice. Thyroid scintigraphy, cross-sectional imaging, and cytological or histopathological examination may aid in establishing the diagnosis. The management strategy depends primarily on the anatomical location and size of the lesion, associated symptoms, thyroid function, and the presence or suspicion of malignancy. Most cases of ETT are located in the tongue base or along the route of thyroglossal duct descent. Rare cases of ETT are located in other parts of the body, such as the neck, trachea, mediastinum, heart, esophagus, diaphragm, adrenal gland, buccal region, and even the infratemporal fossa. As a result, it may cause local symptoms such as dysphagia, dysphonia, and airway obstruction.^[1,2]^ When ETT coexists with a normally located thyroid gland, thyroid scintigraphy may demonstrate a focus of radioiodine uptake outside the normal thyroid bed in addition to tracer uptake within the orthotopic gland anterior to the trachea. By contrast, the orthotopic thyroid gland is absent in patients with complete thyroid ectopia. However, small ectopic lesions may not always be detected by scintigraphy. When the orthotopic thyroid gland appears normal and thyroid function is preserved, ETT may therefore be clinically overlooked or misdiagnosed as another type of neck mass.

Thyroid scintigraphy cannot detect all ETT because visualization depends on sufficient functional tracer-trapping activity. The uptake and retention of ^123^I depend on iodide trapping and subsequent organification, whereas ^99m^Tc-pertechnetate is trapped by thyroid cells but is not organified. Consequently, nonfunctioning or poorly functioning ectopic thyroid remnants, including cystic, atrophic, or fibrotic lesions, may demonstrate little or no radiotracer uptake. Tracer visualization may also be affected by lesion perfusion. Ectopic foci with an inadequate arterial supply may receive insufficient tracer during the imaging period. In addition, small lesions or lesions with a low count density may fall below the spatial resolution of the imaging system or be obscured by background activity, particularly in the neck, upper mediastinum, or sublingual region. Physiological uptake of ^99^mTc-pertechnetate in adjacent structures, such as the salivary glands, may further obscure ectopic foci. Low or suppressed thyroid-stimulating hormone levels, including those resulting from exogenous thyroid hormone therapy, may also reduce tracer uptake. Furthermore, lesions at unusual anatomical sites may lie outside the standard acquisition field, while signals from deeply located lesions may be attenuated by overlying bone or soft tissue. Therefore, a negative or equivocal scintigraphic result does not exclude ETT; it indicates only that no sufficiently active, adequately perfused, and detectable focus was identified within the imaging field under the specific acquisition conditions.^[3,4]^ Complementary imaging modalities, including color Doppler ultrasonography, contrast-enhanced computed tomography, computed tomography angiography, magnetic resonance imaging, and magnetic resonance angiography, may be required for anatomical characterization. Although these modalities may not always provide a definitive diagnosis, they can demonstrate the lesion’s similarity to the orthotopic thyroid gland and delineate its vascularity, feeding arteries, venous drainage, and relationships with adjacent structures.

The differential diagnosis of a nonfunctioning neck lesion suspected to contain thyroid tissue depends largely on its anatomical location. For a midline neck lesion, a thyroglossal duct cyst and a prominent thyroid pyramidal lobe should be considered, while benign or malignant lesions arising within ETT may further complicate the diagnosis. Color Doppler ultrasound is a useful noninvasive method for evaluating the echotexture and vascularity of both the orthotopic thyroid gland and the ectopic lesion. However, although sonographic similarity to the orthotopic thyroid gland may suggest thyroid origin, it does not provide definitive confirmation. A focus of thyroid tissue in the lateral neck coexisting with a normal orthotopic gland may mimic metastatic differentiated thyroid carcinoma. Distinguishing benign ETT from cervical lymph node metastasis of papillary thyroid carcinoma is therefore crucial.^[5,6]^ From an embryological view, benign ectopic tissue is most commonly located in the midline along the path of thyroid descent. When tissue is obtained by fine-needle aspiration or surgical excision, the presence of benign thyroid follicles without cytological or architectural features of malignancy supports the diagnosis of benign ETT.^[7]^ Positive immunohistochemical staining for thyroglobulin confirms thyroid follicular differentiation but cannot, by itself, distinguish benign ETT from metastatic differentiated thyroid carcinoma.

In the present case, a persistent thyroglossal duct and thyroglossal duct cyst were considered unlikely because of the lateral location of the lesion and the absence of a cystic or duct-like structure. Iatrogenic implantation was excluded based on the absence of previous neck surgery or intervention. No suspicious lesion was identified in the orthotopic thyroid gland on Color Doppler ultrasonography. Together with the benign histopathological findings and the absence of recurrence during the 5-year follow-up, this finding made metastasis from an occult orthotopic thyroid carcinoma unlikely. Fiberoptic laryngoscopy and contrast-enhanced CT revealed no primary lesion in the upper aerodigestive tract. Other hypervascular lesions of the carotid space, including carotid body tumors and vagal paragangliomas, should also be considered. Although these lesions are similarly hypervascular, they can usually be differentiated by their anatomical locations and characteristic patterns of vascular displacement on CT angiography or MR angiography. A carotid body tumor typically splays the internal and external carotid arteries at the carotid bifurcation, producing the characteristic Lyre or goblet sign, whereas a vagal paraganglioma usually displaces both arteries together in an anteromedial direction. Schwannomas and branchial cleft cysts generally demonstrate substantially less internal vascularity.

The thyroid gland is a highly vascular organ. Its arterial supply is derived primarily from the bilateral superior and inferior thyroid arteries, although anatomical variations in the origins and courses of these vessels have been documented.^[8]^ Anomalous arterial supply to the orthotopic thyroid gland and distinct vascular pedicles supplying ETT have also been described; however, the available evidence is limited to a small number of case reports.^[9–11]^ Vessels accompanying thyroid tissue during its embryological descent have also been reported.^[12,13]^ Further evidence is required to determine whether a characteristic feeding or draining vascular pattern could serve as an imaging clue for the diagnosis of ETT. In the present case, intraoperative examination revealed that the ETT was supplied by a distinct arterial branch arising directly from the external carotid artery near the carotid bifurcation rather than from the superior thyroid artery. The artery also gave off smaller branches to the SHMs along its descending course. Venous drainage occurred through a short trunk that emptied directly into the IJV. This vascular arrangement partially resembled that of the orthotopic thyroid gland. These findings suggest that ETT may develop a distinct local vascular network. The vascular arrangement observed in this case may represent an an interesting anatomical feature that could be considered during imaging assessment of ETT; however, its diagnostic significance remains uncertain and requires confirmation in additional cases.

In addition to the distinctive vascular anatomy, a cluster of small lymphoid nodules was identified adjacent to the ETT. These nodules were located between the lateral margin of the SHMs and the anterior border of the sternocleidomastoid muscle. Pathologically, the lymphoid tissue appeared to be in an undeveloped status without obvious lymphatic hyperplasia. Their clustered arrangement and gross appearance differed from those of the lymph nodes distributed across ipsilateral cervical levels II to IV. Histopathological examination demonstrated nonhyperplastic lymphoid tissue without prominent reactive hyperplasia or lymphoid follicle formation. Lymphoid tissue was also observed within and adjacent to the ETT, enclosed within the same connective-tissue capsule. The biological significance of this finding remains uncertain. The close spatial relationship between the ETT and the lymphoid aggregates raises the possibility of a developmental association. This may suggest that the development and descent of the thyroid primordium are likely accompanied by some lymphoid tissue, which does not accord with the long-believed classic theory. In that theory, the thyroid gland belongs to the endoderm, and the lymph node belongs to the mesoderm; the thyroid gland and lymph nodes just meet at the right place at a certain period of the embryonic development phase. But the classic theory could not convincingly explain why the lymphoid tissue occurred in the thyroid gland, as shown in Figure 2C. If we hypothesize that the descent of the thyroid gland and lymphatic tissue around the thyroid gland, such as those in the central compartment zone, take place and accompany each other at the same time, it can better explain why lymphatic tissue was present in the thyroid gland and why lymph nodes in the central compartment zone were the sentinel nodes of thyroid cancer. This case also adds some more insight into lymph node metastasis and corresponding dissection strategy in the central compartment zone of thyroid carcinoma, whose embryological basis of lymphatic metastasis has not yet been fully clarified. However, evidence from a single case is insufficient to demonstrate concurrent migration of the thyroid primordium and lymphoid tissue during embryogenesis. Alternative explanations, including incidental adjacent cervical lymph nodes, acquired lymphoid aggregates, and ectopic thymic tissue, should also be considered. Further histopathological characterization, including immunohistochemical analysis, together with additional clinicopathological cases and anatomical studies, is required to clarify the origin and significance of the associated lymphoid tissue.

In summary, a diagnosis of ETT in the carotid sheath was established by integrating clinical findings, imaging, operative observations, and histopathology. This case demonstrates a distinctive vascular pattern, including a feeding artery arising directly from the external carotid artery and a short venous trunk draining into the IJV. The case also documents the unusual presence of lymphoid aggregates within and adjacent to the ETT. Further case series, detailed histopathological examinations, and immunohistochemical studies are needed to determine whether this vascular pattern is reproducible and to clarify the nature and origin of the associated lymphoid tissue. However, the findings from a single case are insufficient to establish a new diagnostic criterion or embryological mechanism.

## Acknowledgments

We thank the patient for granting permission to publish this information.

## Author contributions

**Conceptualization:** Wen Li.

**Data curation:** Yan Gao.

**Project administration:** Liu Yang.

**Resources:** Wen Li.

**Writing – original draft:** Min Chen.
